# 1-(5-Chloro-2,4-dihydroxy­phen­yl)-2-(4-ethoxy­phen­yl)ethanone

**DOI:** 10.1107/S1600536808030699

**Published:** 2008-09-27

**Authors:** Nigel P. Botting, Alexandra M. Z. Slawin, Qingzhi Zhang

**Affiliations:** a Department of Chemistry, University of St Andrews, St Andrews KY16 9ST, Scotland

## Abstract

The structure of the title compound, C_16_H_15_ClO_4_, contains aryl rings which are inclined by 75.6 (1)° to each other. It displays intra­molecular O—H⋯O hydrogen bonding between the 2-hydr­oxy and carbonyl groups, forming a six-membered ring. Furthermore, the 4-hydr­oxy group, acting as a hydrogen-bond donor, is bound to the O atom of the 2-hydr­oxy group of another mol­ecule.

## Related literature

For related literature, see: Anderson & Garner (1997 ▶); Fokialakis *et al.* (2004 ▶); Papoutsi *et al.* (2007 ▶); Anthony (2002 ▶); Barnes (1998 ▶); Barnes & Peterson (1995 ▶); Dixon & Ferreira (2002 ▶); Greenwood *et al.* (2000 ▶); Setchell (1998 ▶); Whalley *et al.* (2000 ▶). For a related structure, see: Arumugan *et al.* (2007 ▶).
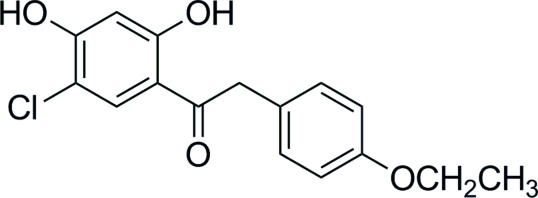


## Experimental

### 

#### Crystal data

C_16_H_15_ClO_4_
*M* *_r_* = 306.73
Monoclinic, 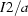

*a* = 19.255 (6) Å
*b* = 4.6454 (15) Å
*c* = 31.109 (11) Å
β = 90.519 (7)°
*V* = 2782.5 (16) Å^3^
*Z* = 8
Mo *K*α radiation
μ = 0.29 mm^−1^
*T* = 125 (2) K
0.11 × 0.03 × 0.03 mm

#### Data collection

Bruker SMART diffractometer
Absorption correction: multi-scan (*SADABS*; Bruker, 2001 ▶) *T*_min_ = 0.983, *T*_max_ = 0.997
8448 measured reflections
2515 independent reflections
1401 reflections with *I* > 2σ(*I*)
*R*_int_ = 0.091

#### Refinement

*R*[*F* ^2^ > 2σ(*F* ^2^)] = 0.049
*wR*(*F* ^2^) = 0.101
*S* = 0.89
2515 reflections
199 parameters
2 restraints
H atoms treated by a mixture of independent and constrained refinement
Δρ_max_ = 0.28 e Å^−3^
Δρ_min_ = −0.27 e Å^−3^

### 

Data collection: *SMART* (Bruker, 2001 ▶); cell refinement: *SMART*; data reduction: *SAINT* (Bruker, 2001 ▶); program(s) used to solve structure: *SHELXTL* (Sheldrick, 2008 ▶); program(s) used to refine structure: *SHELXTL*; molecular graphics: *SHELXTL*; software used to prepare material for publication: *SHELXTL*.

## Supplementary Material

Crystal structure: contains datablocks I, global. DOI: 10.1107/S1600536808030699/wn2279sup1.cif

Structure factors: contains datablocks I. DOI: 10.1107/S1600536808030699/wn2279Isup2.hkl

Additional supplementary materials:  crystallographic information; 3D view; checkCIF report

## Figures and Tables

**Table 1:** Hydrogen-bond geometry (Å, °)

*D*—H⋯*A*	*D*—H	H⋯*A*	*D*⋯*A*	*D*—H⋯*A*
O2—H2O⋯O7	0.98 (1)	1.71 (3)	2.542 (3)	141 (3)
O4—H4O⋯O2^i^	0.98 (1)	1.821 (9)	2.784 (3)	167 (3)

Symmetry code: (i) 
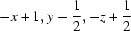
.

**Fig. 1.**
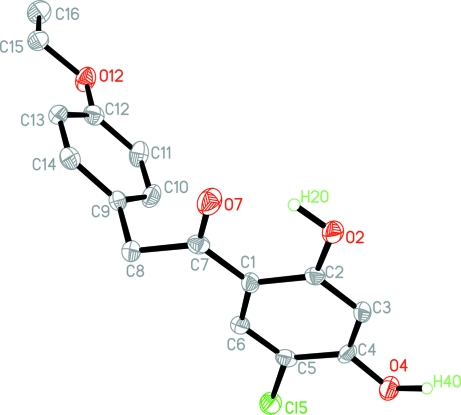
The molecular structure of the title compound, with displacement ellipsoids drawn at the 50% probability level. Carbon-bound H atoms have been omitted; H atoms bonded to oxygen are represented as spheres of arbitrary radius.

**Fig. 2.**
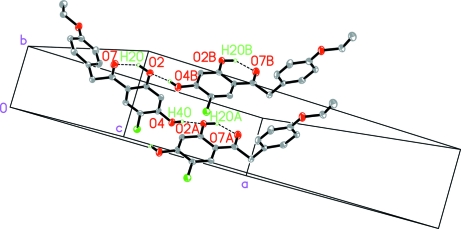
A packing diagram, with hydrogen bonding interactions indicated as dashed lines. Hydrogen atoms not involved in hydrogen bonding have been omitted.

## supplementary crystallographic information

### Comment

Phytoestrogens, in particular the soy isoflavones such as daidzein and genistein, have positive impact on human health (Setchell, 1998; Barnes, 1998 and Dixon & Ferreira, 2002). High consumption of soy products has been associated with a low incidence of hormone-dependent cancers (Barnes & Peterson, 1995), the symptom alleviation of menopause (Greenwood *et al.*, 2000) and protection against osteoporosis (Anderson & Garner, 1997) as well as cardiovascular disease (Anthony, 2002). Consequently, there is growing interest in using phytoestrogens and synthetic derivatives for the chemoprevention and therapy of these diseases.

One of the synthetic routes to daidzein and its derivatives is *via* the Freidel-Crafts reaction of resorcinol and phenylacetic acid catalysed by boron trifluoride etherate (Whalley, *et al.*, 2000), giving the deoxybenzoin intermediate. In the preparation of 5-chlorodaidzein, 1-(5-chloro-2,4-dihydroxyphenyl)-2-(4-hydroxyphenyl)-ethanone (2) was obtained (yield 77%) from the coupling of 5-cholororesorcinol with 4-hydroxyphenylacetic acid in boron trifluoride etherate. Surprisingly, the title compound, 1-(5-chloro-2,4-dihydroxyphenyl)-2-(4-ethoxyphenyl)-ethanone (1), was also isolated in small amount (5% yield). Boron trifluoride proves to be such a strong Lewis acid, it not only catalyses the Freidel-Crafts reaction, but also activates the diethyl ether and makes it an electrophile. From the position of ethylation, it can be concluded that (1) is formed from the attack of (2) *via* 4-hydroxyphenyl to the α-position of the actived diethyl ether, because the 4-OH of the benzyl ring is more nucleophilic than the 2-OH and the 4-OH in the other phenyl ring which bears the electron-withdrawing carbonyl group. (1) has been previously synthesized as the major product from 5-chlororesorcinol and 4-ethoxyphenylacetic acid (Fokialakis *et al.* 2004). The deoxybenzoin shows some estrogenic activity like daidzein (Fokialakis *et al.*, 2004; Papoutsi *et al.*, 2007).

The molecular structure of (1) is conformationally similar to that of deoxyanisoin, MeOC_6_H_4_C(O)CH_2_C_6_H_4_OMe. (Arumugan *et al.* 2007) with statistically invariant C═O and C—C bond lengths and very similar backbone torsion angles, though C4—O4 in 1 appears to be marginally shorter at 1.347 (3) Å than in deoxyanisoin at 1.378 (1) Å; this may be a consequence of the neighbouring chloro substituent in (1).

### Experimental

Boron trifluoride diethyl etherate (7.7 ml, 60.7 mmol) was added to a mixture of resorcinol (4.4 g, 30.4 mmol) and 3-chloro-4-hydroxyphenylacetic acid (4.63 g, 30.4 mmol) under a nitrogen atmosphere. The mixture was heated to reflux for 5 h, cooled to room temperature, and saturated aqueous sodium acetate (50 ml) and aqueous sodium hydrogen carbonate (40 ml) added sequentially. The mixture was extracted with diethyl ether (3 × 50 ml), dried MgSO_4_ and the solvent removed at reduced pressure.Column chromatography on silica, with hexane/ethyl acetate (1:1) as eluant, gave the title compound (1) (0.47 g, 5%). ^1^H NMR and ^13^C NMR (CDCl_3_) of (1) as well as MS were in agreement with that in the literature (Fokialakis *et al.*, 2004).

### Refinement

All carbon-bound H-atoms were included in calculated positions (C—H distances are 0.98 Å for methyl H atoms, 0.99 Å for methylene H and 0.95 Å for aryl H atoms) and were refined as riding atoms with U_iso_(H) = xU_eq_(parent atom), where x = 1.2  for methylene and aryl H atoms, 1.5 for methyl H atoms. The OH hydrogen atoms were located in a difference map and refined isotropically, subject to a distance restraint, 0.98 (1) Å.

### Crystal data

**Table:**

C_16_H_15_ClO_4_	*F*(000) = 1280
*M**_r_* = 306.73	*D*_x_ = 1.464 Mg m^−^^3^
Monoclinic, *I*2/*a*	Mo *K*α radiation, λ = 0.71073 Å
*a* = 19.255 (6) Å	Cell parameters from 306 reflections
*b* = 4.6454 (15) Å	θ = 12–28°
*c* = 31.109 (11) Å	µ = 0.29 mm^−^^1^
β = 90.519 (7)°	*T* = 125 K
*V* = 2782.5 (16) Å^3^	Prism, colourless
*Z* = 8	0.11 × 0.03 × 0.03 mm

### Data collection

**Table:**

Bruker SMART diffractometer	2515 independent reflections
Radiation source: fine-focus sealed tube	1401 reflections with *I* > 2σ(*I*)
graphite	*R*_int_ = 0.091
Detector resolution: 0.83 pixels mm^-1^	θ_max_ = 25.4°, θ_min_ = 2.1°
φ and ω scans	*h* = −22→23
Absorption correction: multi-scan (SADABS; Bruker, 2001)	*k* = −5→5
*T*_min_ = 0.983, *T*_max_ = 0.997	*l* = −37→31
8448 measured reflections	

### Refinement

**Table:**

Refinement on *F*^2^	Primary atom site location: structure-invariant direct methods
Least-squares matrix: full	Secondary atom site location: difference Fourier map
*R*[*F*^2^ > 2σ(*F*^2^)] = 0.049	Hydrogen site location: inferred from neighbouring sites
*wR*(*F*^2^) = 0.101	H atoms treated by a mixture of independent and constrained refinement
*S* = 0.89	*w* = 1/[σ^2^(*F*_o_^2^) + (0.0438*P*)^2^] where *P* = (*F*_o_^2^ + 2*F*_c_^2^)/3
2515 reflections	(Δ/σ)_max_ = 0.033
199 parameters	Δρ_max_ = 0.28 e Å^−^^3^
2 restraints	Δρ_min_ = −0.27 e Å^−^^3^

### Special details

**Table:**

Geometry. All e.s.d.'s (except the e.s.d. in the dihedral angle between two l.s. planes) are estimated using the full covariance matrix. The cell e.s.d.'s are taken into account individually in the estimation of e.s.d.'s in distances, angles and torsion angles; correlations between e.s.d.'s in cell parameters are only used when they are defined by crystal symmetry. An approximate (isotropic) treatment of cell e.s.d.'s is used for estimating e.s.d.'s involving l.s. planes.
Refinement. Refinement of *F*^2^ against ALL reflections. The weighted *R*-factor *wR* and goodness of fit *S* are based on *F*^2^, conventional *R*-factors *R* are based on *F*, with *F* set to zero for negative *F*^2^. The threshold expression of *F*^2^ > σ(*F*^2^) is used only for calculating *R*-factors(gt) *etc*. and is not relevant to the choice of reflections for refinement. *R*-factors based on *F*^2^ are statistically about twice as large as those based on *F*, and *R*- factors based on ALL data will be even larger.

### Fractional atomic coordinates and isotropic or equivalent isotropic displacement parameters (Å^2^)

**Table:**

	*x*	*y*	*z*	*U*_iso_*/*U*_eq_	
C1	0.33978 (15)	0.7593 (6)	0.18759 (10)	0.0179 (7)	
C2	0.39148 (16)	0.8676 (6)	0.21571 (10)	0.0178 (8)	
O2	0.37615 (11)	1.0672 (4)	0.24646 (7)	0.0217 (5)	
H2O	0.3277 (6)	1.130 (8)	0.2439 (13)	0.076 (14)*	
C3	0.45909 (15)	0.7723 (6)	0.21336 (10)	0.0189 (7)	
H3A	0.4931	0.8474	0.2326	0.023*	
C4	0.47810 (16)	0.5683 (6)	0.18323 (10)	0.0193 (8)	
O4	0.54308 (11)	0.4628 (5)	0.18053 (7)	0.0258 (6)	
H4O	0.5655 (16)	0.516 (8)	0.2078 (6)	0.064 (13)*	
C5	0.42808 (16)	0.4638 (6)	0.15414 (10)	0.0195 (8)	
Cl5	0.45154 (4)	0.21806 (16)	0.11487 (3)	0.0265 (3)	
C6	0.36084 (16)	0.5567 (6)	0.15684 (10)	0.0192 (8)	
H6A	0.3273	0.4817	0.1373	0.023*	
C7	0.26827 (16)	0.8586 (6)	0.19047 (10)	0.0193 (8)	
O7	0.25248 (11)	1.0457 (5)	0.21737 (7)	0.0295 (6)	
C8	0.21213 (15)	0.7476 (6)	0.16079 (10)	0.0234 (8)	
H8A	0.2267	0.5604	0.1486	0.028*	
H8B	0.1692	0.7156	0.1774	0.028*	
C9	0.19729 (16)	0.9550 (6)	0.12476 (10)	0.0187 (8)	
C10	0.24738 (17)	1.0299 (7)	0.09503 (11)	0.0258 (8)	
H10A	0.2925	0.9486	0.0974	0.031*	
C11	0.23309 (16)	1.2204 (7)	0.06193 (10)	0.0270 (8)	
H11A	0.2681	1.2687	0.0419	0.032*	
C12	0.16775 (17)	1.3401 (7)	0.05814 (11)	0.0239 (8)	
C13	0.11677 (16)	1.2740 (6)	0.08744 (10)	0.0234 (8)	
H13A	0.0719	1.3582	0.0851	0.028*	
C14	0.13229 (16)	1.0809 (6)	0.12065 (10)	0.0222 (8)	
H14A	0.0974	1.0349	0.1409	0.027*	
O12	0.15829 (10)	1.5204 (5)	0.02307 (7)	0.0293 (6)	
C15	0.09030 (16)	1.6385 (7)	0.01654 (11)	0.0271 (9)	
H15A	0.0773	1.7614	0.0412	0.033*	
H15B	0.0556	1.4824	0.0136	0.033*	
C16	0.09290 (17)	1.8138 (7)	−0.02397 (11)	0.0336 (9)	
H16A	0.0472	1.8999	−0.0296	0.050*	
H16B	0.1055	1.6894	−0.0481	0.050*	
H16C	0.1276	1.9667	−0.0207	0.050*	

### Atomic displacement parameters (Å^2^)

**Table:**

	*U*^11^	*U*^22^	*U*^33^	*U*^12^	*U*^13^	*U*^23^
C1	0.0173 (17)	0.0189 (17)	0.0175 (18)	−0.0018 (14)	−0.0035 (14)	0.0016 (14)
C2	0.0209 (19)	0.0137 (16)	0.019 (2)	−0.0004 (14)	−0.0014 (15)	0.0023 (13)
O2	0.0231 (13)	0.0224 (12)	0.0195 (13)	0.0028 (10)	−0.0057 (11)	−0.0029 (10)
C3	0.0188 (17)	0.0197 (17)	0.0181 (18)	−0.0015 (15)	−0.0058 (14)	0.0000 (15)
C4	0.0155 (17)	0.0210 (18)	0.021 (2)	0.0020 (15)	−0.0025 (15)	0.0071 (15)
O4	0.0217 (13)	0.0300 (14)	0.0257 (15)	0.0023 (11)	−0.0048 (11)	−0.0025 (11)
C5	0.0269 (19)	0.0146 (17)	0.0170 (19)	−0.0009 (15)	−0.0009 (16)	0.0008 (14)
Cl5	0.0277 (5)	0.0265 (5)	0.0250 (5)	0.0027 (4)	−0.0029 (4)	−0.0061 (4)
C6	0.0208 (18)	0.0156 (17)	0.021 (2)	0.0002 (14)	−0.0031 (15)	−0.0006 (14)
C7	0.0247 (19)	0.0157 (17)	0.017 (2)	−0.0003 (14)	−0.0004 (16)	0.0028 (14)
O7	0.0270 (13)	0.0342 (14)	0.0273 (15)	0.0057 (11)	−0.0066 (11)	−0.0070 (11)
C8	0.0191 (17)	0.0207 (18)	0.030 (2)	−0.0013 (15)	−0.0065 (15)	0.0011 (16)
C9	0.0183 (18)	0.0181 (18)	0.020 (2)	−0.0014 (15)	−0.0037 (16)	−0.0024 (14)
C10	0.0191 (18)	0.031 (2)	0.028 (2)	0.0033 (16)	−0.0058 (17)	−0.0016 (17)
C11	0.0193 (18)	0.038 (2)	0.024 (2)	−0.0013 (17)	0.0011 (15)	−0.0004 (17)
C12	0.029 (2)	0.0213 (18)	0.021 (2)	−0.0015 (15)	−0.0079 (17)	−0.0006 (15)
C13	0.0203 (17)	0.0201 (18)	0.030 (2)	0.0024 (15)	−0.0057 (16)	−0.0024 (16)
C14	0.0222 (19)	0.0230 (18)	0.021 (2)	−0.0043 (15)	0.0019 (16)	−0.0006 (15)
O12	0.0236 (13)	0.0358 (14)	0.0285 (15)	0.0003 (11)	−0.0052 (11)	0.0087 (11)
C15	0.0272 (19)	0.0232 (18)	0.031 (2)	0.0013 (15)	−0.0077 (17)	0.0063 (15)
C16	0.035 (2)	0.035 (2)	0.031 (2)	0.0023 (18)	−0.0047 (18)	0.0049 (17)

### Geometric parameters (Å, °)

**Table:**

C1—C6	1.404 (4)	C9—C14	1.386 (4)
C1—C2	1.412 (4)	C9—C10	1.387 (4)
C1—C7	1.456 (4)	C10—C11	1.384 (4)
C2—O2	1.366 (4)	C10—H10A	0.9500
C2—C3	1.378 (4)	C11—C12	1.380 (4)
O2—H2O	0.9799 (11)	C11—H11A	0.9500
C3—C4	1.385 (4)	C12—C13	1.380 (5)
C3—H3A	0.9500	C12—O12	1.386 (4)
C4—O4	1.347 (3)	C13—C14	1.398 (4)
C4—C5	1.402 (4)	C13—H13A	0.9500
O4—H4O	0.9800 (11)	C14—H14A	0.9500
C5—C6	1.368 (4)	O12—C15	1.432 (4)
C5—Cl5	1.735 (3)	C15—C16	1.502 (4)
C6—H6A	0.9500	C15—H15A	0.9900
C7—O7	1.246 (4)	C15—H15B	0.9900
C7—C8	1.506 (4)	C16—H16A	0.9800
C8—C9	1.504 (4)	C16—H16B	0.9800
C8—H8A	0.9900	C16—H16C	0.9800
C8—H8B	0.9900		
			
C6—C1—C2	117.0 (3)	C14—C9—C8	120.3 (3)
C6—C1—C7	122.2 (3)	C10—C9—C8	122.0 (3)
C2—C1—C7	120.7 (3)	C11—C10—C9	121.5 (3)
O2—C2—C3	117.7 (3)	C11—C10—H10A	119.3
O2—C2—C1	121.3 (3)	C9—C10—H10A	119.3
C3—C2—C1	120.9 (3)	C12—C11—C10	119.8 (3)
C2—O2—H2O	111 (2)	C12—C11—H11A	120.1
C2—C3—C4	120.8 (3)	C10—C11—H11A	120.1
C2—C3—H3A	119.6	C11—C12—C13	120.5 (3)
C4—C3—H3A	119.6	C11—C12—O12	115.1 (3)
O4—C4—C3	122.8 (3)	C13—C12—O12	124.4 (3)
O4—C4—C5	117.8 (3)	C12—C13—C14	118.8 (3)
C3—C4—C5	119.3 (3)	C12—C13—H13A	120.6
C4—O4—H4O	105 (2)	C14—C13—H13A	120.6
C6—C5—C4	119.7 (3)	C9—C14—C13	121.7 (3)
C6—C5—Cl5	120.2 (2)	C9—C14—H14A	119.1
C4—C5—Cl5	120.0 (2)	C13—C14—H14A	119.1
C5—C6—C1	122.2 (3)	C12—O12—C15	117.2 (3)
C5—C6—H6A	118.9	O12—C15—C16	106.8 (3)
C1—C6—H6A	118.9	O12—C15—H15A	110.4
O7—C7—C1	119.9 (3)	C16—C15—H15A	110.4
O7—C7—C8	118.2 (3)	O12—C15—H15B	110.4
C1—C7—C8	121.9 (3)	C16—C15—H15B	110.4
C9—C8—C7	111.6 (2)	H15A—C15—H15B	108.6
C9—C8—H8A	109.3	C15—C16—H16A	109.5
C7—C8—H8A	109.3	C15—C16—H16B	109.5
C9—C8—H8B	109.3	H16A—C16—H16B	109.5
C7—C8—H8B	109.3	C15—C16—H16C	109.5
H8A—C8—H8B	108.0	H16A—C16—H16C	109.5
C14—C9—C10	117.7 (3)	H16B—C16—H16C	109.5
			
C6—C1—C2—O2	179.5 (3)	C2—C1—C7—C8	179.8 (3)
C7—C1—C2—O2	0.3 (4)	O7—C7—C8—C9	78.3 (4)
C6—C1—C2—C3	−1.4 (4)	C1—C7—C8—C9	−100.0 (3)
C7—C1—C2—C3	179.4 (3)	C7—C8—C9—C14	−117.3 (3)
O2—C2—C3—C4	179.4 (3)	C7—C8—C9—C10	61.9 (4)
C1—C2—C3—C4	0.2 (5)	C14—C9—C10—C11	−0.9 (5)
C2—C3—C4—O4	−178.1 (3)	C8—C9—C10—C11	179.8 (3)
C2—C3—C4—C5	1.6 (5)	C9—C10—C11—C12	0.1 (5)
O4—C4—C5—C6	177.4 (3)	C10—C11—C12—C13	0.8 (5)
C3—C4—C5—C6	−2.3 (5)	C10—C11—C12—O12	−177.9 (3)
O4—C4—C5—Cl5	−2.6 (4)	C11—C12—C13—C14	−0.8 (5)
C3—C4—C5—Cl5	177.6 (2)	O12—C12—C13—C14	177.8 (3)
C4—C5—C6—C1	1.1 (5)	C10—C9—C14—C13	0.9 (5)
Cl5—C5—C6—C1	−178.8 (2)	C8—C9—C14—C13	−179.8 (3)
C2—C1—C6—C5	0.7 (5)	C12—C13—C14—C9	−0.1 (5)
C7—C1—C6—C5	179.9 (3)	C11—C12—O12—C15	176.9 (3)
C6—C1—C7—O7	−177.6 (3)	C13—C12—O12—C15	−1.7 (4)
C2—C1—C7—O7	1.5 (4)	C12—O12—C15—C16	−177.7 (3)
C6—C1—C7—C8	0.6 (4)		

### Hydrogen-bond geometry (Å, °)

**Table:**

*D*—H···*A*	*D*—H	H···*A*	*D*···*A*	*D*—H···*A*
O2—H2O···O7	0.98 (1)	1.71 (3)	2.542 (3)	141 (3)
O4—H4O···O2^i^	0.98 (1)	1.82 (1)	2.784 (3)	167 (3)

Symmetry codes: (i) −*x*+1, *y*−1/2, −*z*+1/2.
